# Supplementary material for: 4-Hydroxycoumarin Exhibits Antinociceptive and Anti-Inflammatory Effects Through Cytokine Modulation: An Integrated In Silico and In Vivo Study
Source: Int J Mol Sci. 2025 Mar 19;26(6):2788. doi: 10.3390/ijms26062788 (PMC11943305; doi:10.3390/ijms26062788)

4-Hydroxycoumarin Exhibits Antinociceptive and Anti-Inflammatory Effects Through Cytokine Modulation: An Integrated In Silico and In Vivo Study

Figure S1. Amino acid interactions formed between 4-HC and celecoxib with COX-2.

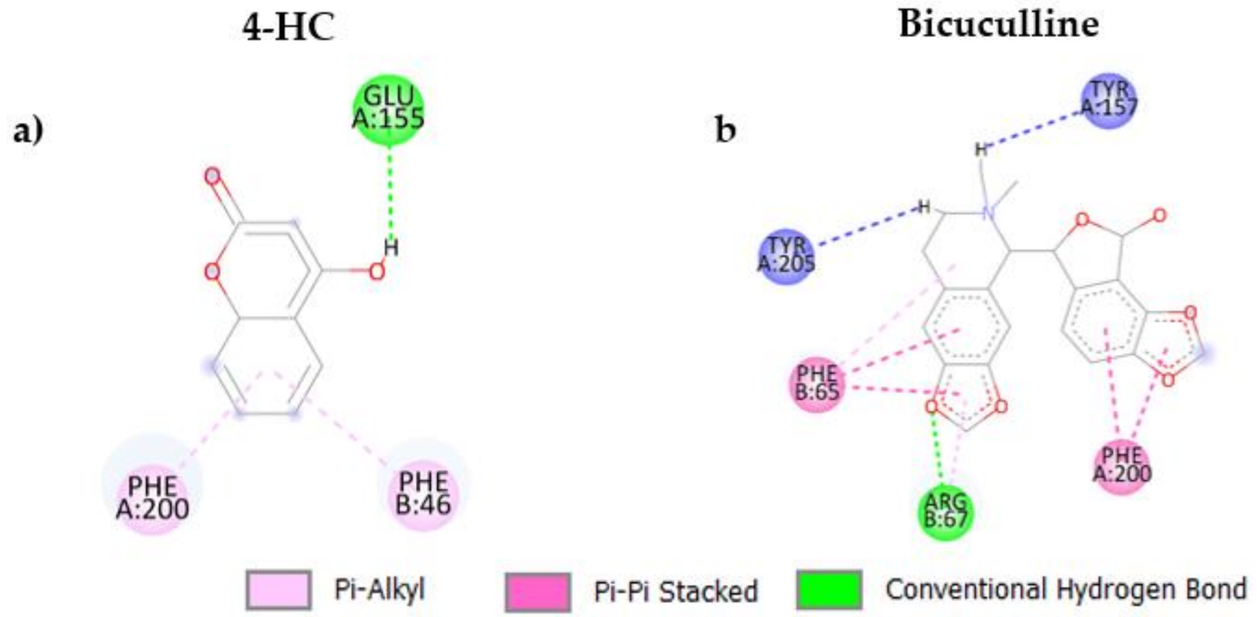

Figure S2. Amino acid interactions formed between 4-HC and bicuculline with $GABA_A$.

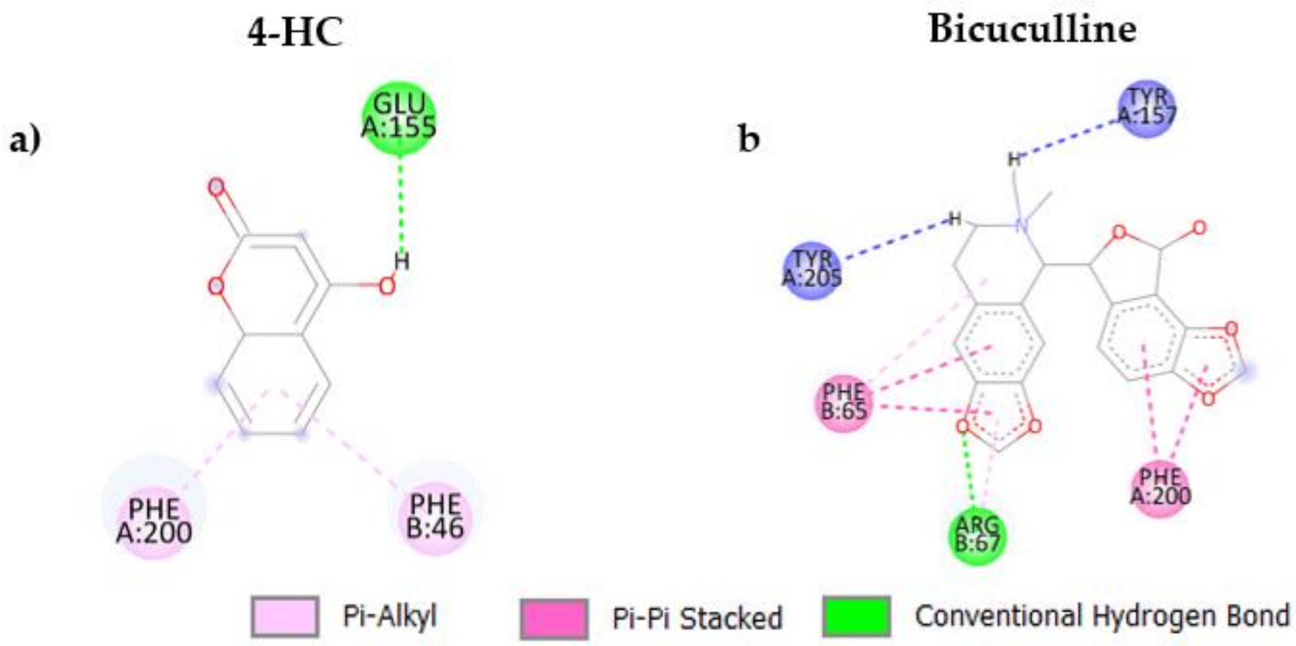

Figure S3. Amino acid interactions formed between 4-HC and 7-Nitroindazole with iNOS.

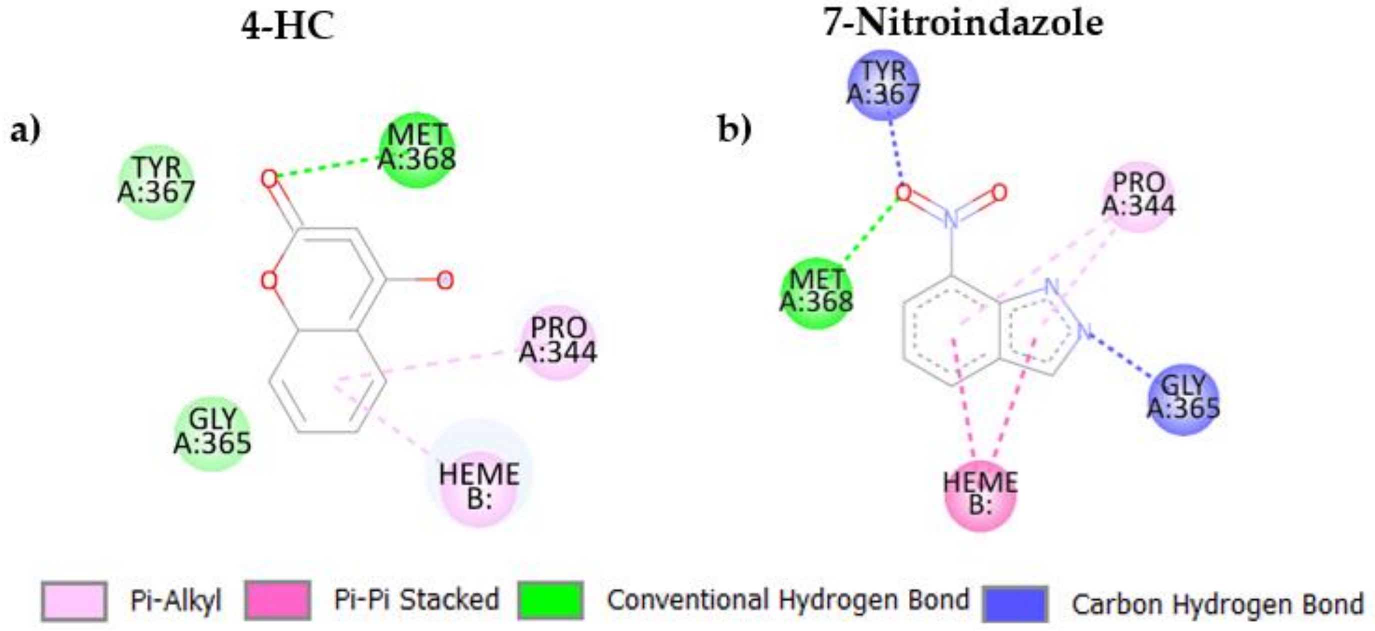

Figure S4. Amino acid interactions formed between 4-HC and dexamethasone with NFκB.

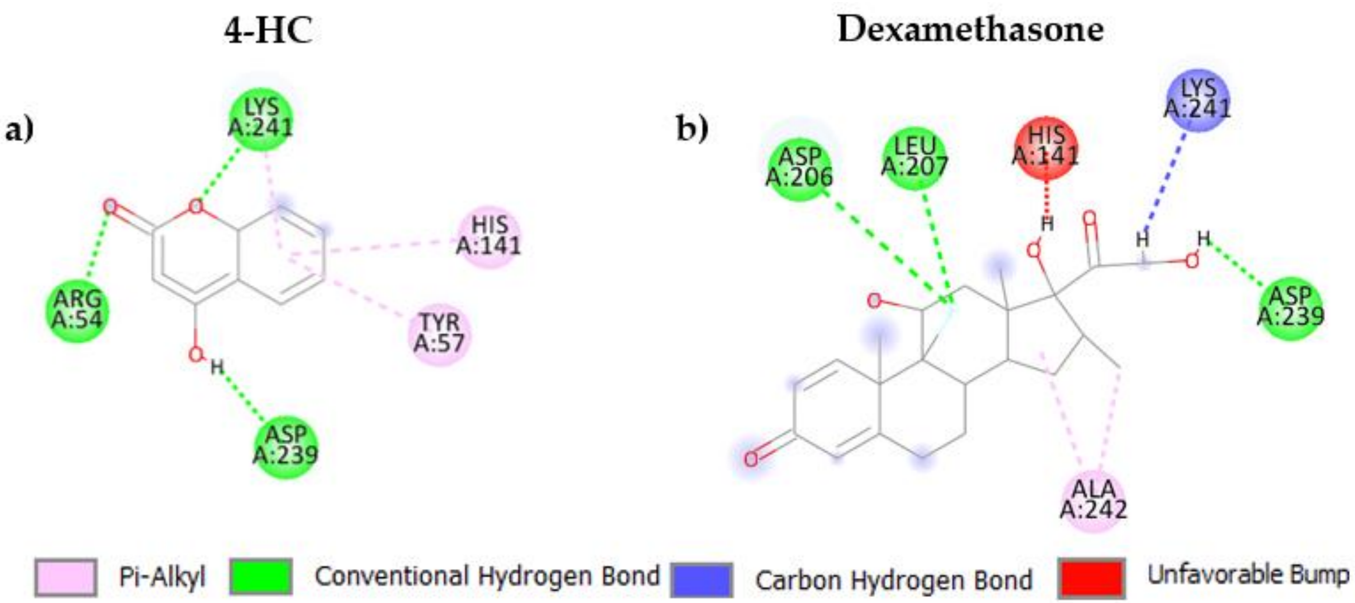

Figure S5. Amino acid interactions formed between 4-HC and morphine with μ-opioid receptor.

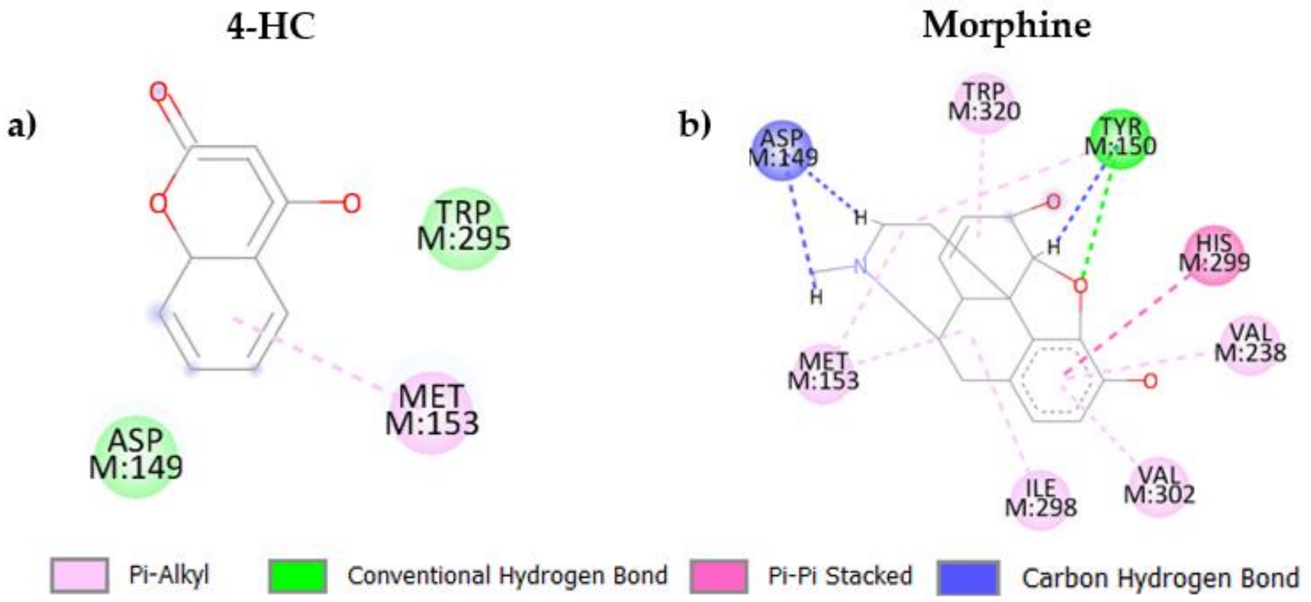

Figure S6. Amino acid interactions formed between 4-HC and sketamine with NMDA$_R$.

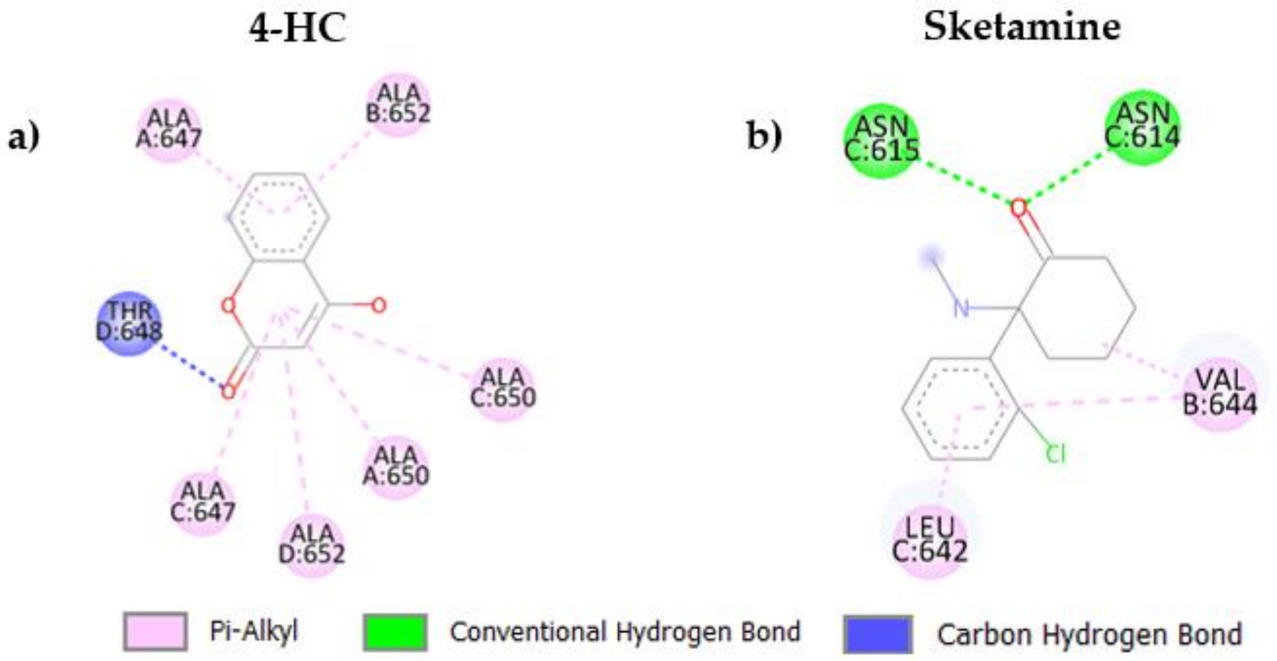

Figure S7. Amino acid interactions formed between 4-HC and capsazepine with TRPV1.

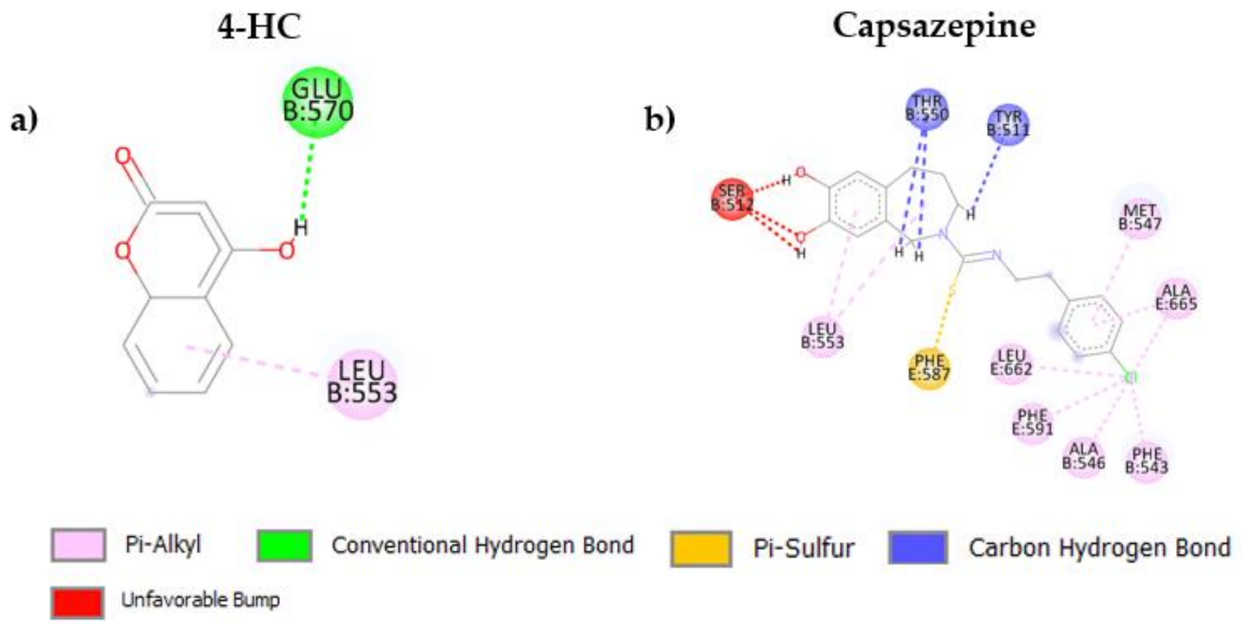

Supplement: Supplementary file 1 [file ijms-26-02788-s001.zip › ijms-3501004-supplementary.pdf]
